# Wastewater treatment works change the intestinal microbiomes of insectivorous bats

**DOI:** 10.1371/journal.pone.0247475

**Published:** 2021-03-03

**Authors:** Calvin Mehl, M. Corrie Schoeman, Tomasz J. Sanko, Carlos Bezuidenhout, Charlotte M. S. Mienie, Wolfgang Preiser, Dalene Vosloo

**Affiliations:** 1 School of Life Sciences, University of KwaZulu-Natal, Durban, South Africa; 2 Unit for Environmental Sciences and Management, North-West University, Potchefstroom, South Africa; 3 Division of Medical Virology, Department of Pathology, Faculty of Medicine and Health Sciences, Stellenbosch University, Tygerberg, Cape Town, South Africa; 4 National Health Laboratory Service (NHLS), Tygerberg Hospital, Tygerberg, South Africa; 5 Centre for Functional Biodiversity, School of Life Sciences, University of KwaZulu-Natal, Durban, South Africa; University of Illinois at Chicago, UNITED STATES

## Abstract

Mammals, born with a near-sterile intestinal tract, are inoculated with their mothers’ microbiome during birth. Thereafter, extrinsic and intrinsic factors shape their intestinal microbe assemblage. Wastewater treatment works (WWTW), sites synonymous with pollutants and pathogens, receive influent from domestic, agricultural and industrial sources. The high nutrient content of wastewater supports abundant populations of chironomid midges (Diptera), which transfer these toxicants and potential pathogens to their predators, such as the banana bat *Neoromicia nana* (Vespertilionidae), thereby influencing their intestinal microbial assemblages. We used next generation sequencing and 16S rRNA gene profiling to identify and compare intestinal bacteria of *N*. *nana* at two reference sites and two WWTW sites. We describe the shared intestinal microbiome of the insectivorous bat, *N*. *nana*, consisting of seven phyla and eleven classes. Further, multivariate analyses revealed that location was the most significant driver (sex, body size and condition were not significant) of intestinal microbiome diversity. Bats at WWTW sites exhibited greater intestinal microbiota diversity than those at reference sites, likely due to wastewater exposure, stress and/or altered diet. Changes in their intestinal microbiota assemblages may allow these bats to cope with concomitant stressors.

## Introduction

In mammals, the intestinal microbiome is derived from the mother during gestation and birth [1, 2]. Thereafter, factors such as environmental conditions, social interaction, diet and host physiology shape their microbial assemblage [1, 3, 4]. The core microbiome, consisting of all microbiota that perform a critical function and are common across spatio-temporal scales [5], is believed to play key roles in ecosystem functioning [6]. These shared microbiota, which make up only a portion of the diverse microbiota inhabiting a hosts gastrointestinal tract, are not necessary shared by all individuals of a species, but rather by subpopulations [7].

In general, intestinal bacteria play key roles in behaviour [8], immune function [9], nutrient absorption [10, 11], storage of fats [12], and detoxification of ingested metals and other pollutants [13, 14]. A high diversity of intestinal microbiota is essential to maintaining the assemblage’s resilience to environmental changes [15]. Further, changes in the intestinal microbiome may significantly reduce the intestinal barrier, thereby exposing the host to infection [16]. Exposure to toxicants and pathogens may also result in dysbiosis of these assemblages, particularly when exposed to these concurrently [17, 18].

Wastewater treatment works (WWTW) receive influent from domestic, agricultural and industrial sources, and hence are one of the most prolific sources of pollution in the urban environment. Wastewater may contain a cocktail of metals [19], pharmaceuticals [20], microbial pathogens [21], natural and synthetic hormones [22], antibiotics [23] and organic chemicals [20]. The nutrient rich waters at and downstream from WWTW favour large numbers of pollutant tolerant [24] insects to thrive [19, 25, 26]. These insects accumulate toxicants (mainly metals, pesticides, polychlorinated biphenyls and polycyclic aromatic hydrocarbons) from sediment and pass them on to their predators (such as insectivorous bats and birds) that are attracted to these sites by the high concentrations of prey [19, 27]. Recent studies have confirmed that bats foraging at WWTW accumulate metals in their tissues [19, 28, 29], leading to increased DNA damage, decreased antioxidant capacity [28, 29] and lesion formation in the liver and kidneys [30]. Untreated or inadequately treated wastewater, released into surrounding ecosystems [31], may expose organisms to bacterial, viral, protozoal, fungal and helminth infections [21] thereby altering the holobiont homeostasis. Thus, WWTW provide a unique environment where predators are exposed to a plethora of concomitant stresses. However, data on the impact of these stresses on predators’ microbiomes are scant.

**Fig 1:**
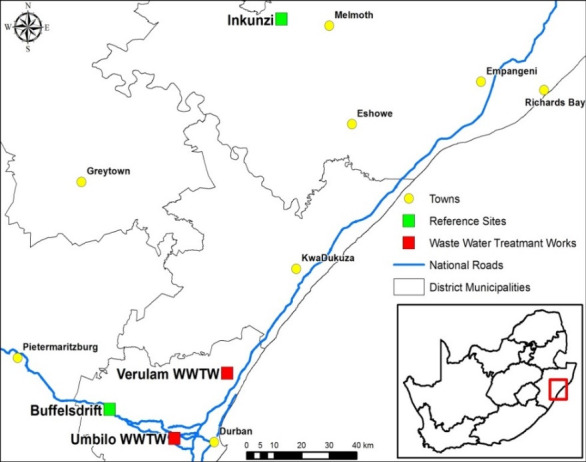
Locality map of wastewater treatment works (WWTW) and reference sites, KwaZulu-Natal, South Africa. Red squares represent WWTWs and green squares represent reference sites. Sites are the same as those used in [36].

Using DNA meta-barcoding of intestinal scrapings, we compared the intestinal microbiota of the insectivorous bat, *Neoromicia nana*, at two WWTW (Verulam and Umbilo) and two reference sites (Buffelsdrift and Inkunzi) in KwaZulu-Natal, South Africa (Fig 1). We describe the shared intestinal microbiota of these individuals and identified the most significant drivers (sex, site, body size and condition) influencing intestinal bacteria assemblages in these bats. Reference sites were situated several kilometres away from WWTW, beyond the expected foraging range of these bats. We predicted a greater diversity of intestinal microbiota in bats at WWTW due to their association with bacterial rich waters at WWTW.

## Materials and methods

### Sampling

Bats were captured using mist nets at WWTW and by hand from roosts at reference sites (Fig 1) during May and June 2015. Individuals were identified to species using a taxonomic key [32]. Non-target animals were released at the capture site. Captured *N*. *nana* bats were sexed and aged (adult or sub-adult) -[33]. Forearm length (to the nearest 0.1 mm) and mass (to the nearest 0.5 g) were measured using calipers and a Pesola scale, respectively. Body condition index (BCI) was calculated as body mass/forearm length [34]. Bats were kept individually in cotton bags overnight. The following morning, bats were euthanised by decapitation while still in torpor in line with AVMA guidelines for obtaining uncontaminated samples [35] Tissues were weighed and frozen in dry ice or liquid nitrogen before storage at -80°C until further analysis. This study formed part of a multidisciplinary collaboration; the protocol was approved by the Animal Research Ethics Committee of the University of KwaZulu-Natal (permit number: 014/015/Animal). Researchers obtained the necessary rabies and HepB vaccinations and wore appropriate PPE (gloves, masks) during all parts of the project.

### DNA extraction and quantification from intestinal scrapings

Using sterilized equipment and working in a laminar flow cabinet, the bat intestines were dissected from the stomach, cut longitudinally and the interior was scraped to remove gut contents. Genomic DNA was extracted from the gut contents using a NucleoSpin® Tissue kit (Macherey-Nagel, Düren, Germany). DNA concentrations were measured using a NanoDrop (Thermo Scientific, Waltham, MA, USA).

### Bacterial 16S rRNA gene sequence amplification, PCR cleanup, quantification and next generation sequencing

Almost complete sequences of the bacterial 16S rRNA gene were amplified using universal 27F (5’-AGAGTTTGATCMTGGCTCAG-3’) and 1492R ((5’-TACCTTGTTACGACTT-3’) primers (Inqaba Biotec, Pretoria, RSA). The PCR mix contained 100–200 ng DNA, 2x KAPA HiFi HotStart ReadyMix (Kapa Biosystems, Wilmington, MA, USA) and 0.5 μM of each primer in a final volume of 10 μL. The PCR thermal cycle started with 2 min denaturation at 98°C followed by 25 cycles of: denaturation at 98°C for 15 sec, annealing at 55°C for 30 sec and elongation at 72°C for 20 sec. The amplification ended with a final elongation step at 72°C for 5 min. This was followed by a nested PCR to increase yield and specificity [37], and targeted the hypervariable V3-V4 region of the bacterial 16S rRNA gene using locus-specific primers 341F and 805R (16S forward primer 5’–TCG TCG GCA GCG TCA GAT GTG TAT AAG AGA CAG CCT ACG GGN GGC WG CAG –3’: 16S reverse primer 5’–GTC TCG TGG GCT CGG AGA TGT GTA TAA GAG ACA GGA CTA CHV GGG TAT CTA ATC C –3’) [38] attached to forward and reverse overhang adapters (Illumina, San Diego, CA, USA). The nested PCR was carried out with 0.5 μL of a 1:50 dilution of the PCR product from the previous step, 2x KAPA HiFi HotStart ReadyMix and 0.4 μM of each primer in a final volume of 25 μL, using the same cycling protocol as in the previous amplification. MiSeq 2 x 300 bp paired-end reads sequencing run was then performed (Illumina MiSeq; Illumina, San Diego, CA, USA). This was followed by de-multiplexing and secondary analyses of the reads using the MiSeq reporter software (Illumina, San Diego, CA, USA) as per the manufacturer’s protocol.

Agencourt AMPure XP beads (Beckman Coulter Genomics, California, USA) were used to clean-up the amplicons obtained from the nested PCR. Thereafter, a PCR reaction attaching dual indexes (Nextera XT Index Kit; Illumina, San Diego, CA, USA) was performed using 5 μL of the PCR amplification product, 5 μL of Illumina Nextera XT Index Primer 1 (N7xx), 5 μL of Nextera XT Index Primer 2 (S5xx), 25 μL of 2x KAPA HiFi HotStart Ready Mix, and 10 μL of PCR-grade water. The PCR cycles were as follows: 95°C for 3 min followed by 8 cycles of denaturation at 95°C for 30 sec, annealing at 55°C for 30 sec, elongation at 72°C for 30 sec and final elongation at 72°C for 5 min. The PCR products were again cleaned up with Agencourt AMPure XP beads (Beckman Coulter Genomics, Brea, CA, USA).

### Sequence analysis

Primers, adapter sequences, reads with a low quality score (less than 15) and short reads (fewer than 25 bp) were removed using Trimmomatic v0.36 [39]. Trimmed sequences were analysed with Quantitative Insights Into Microbial Ecology (QIIMETM, [40]). Forward and reverse reads were merged with PandaSeq [41]. Only sequences equal or longer than 200bp were used with a threshold similarity of 80%. Singletons were removed and open reference OTUs were selected from the Silva 128 database [42] using usearch61 [43]. A single rarefaction filtration step (19688 reads) was performed to reduce bias among samples of unequal numbers of reads and a summarised operational taxonomic unit (OTU) table was constructed.

### Data handling

Statistical analyses were performed using R software v3.2.2 [44]. Normality and homogeneity of variance were tested using Shapiro-Wilk tests and Levene’s tests, respectively. Assumptions for parametric statistics were violated for all data, even after transformation. Therefore, Kruskall-Wallis rank sums were used to compare OTU abundances, forearm length, body mass, diversity indices and BCI between sites, and Wilcoxon signed ranks test to compare OTU abundances, forearm length, body mass and BCI between grouped sites and sexes [45–48]. Dunn’s test was used as the post hoc test for the Kruskall-Wallis rank sums test using the dunn.test package [49] in R. Simpson’s and Shannon-Weiner diversity indices and NMDS were calculated using the vegan package [50]⁠. Relationships between BCI and bacterial diversity at each taxonomic level was determined with Spearman-rank order correlation matrices. A permanova was used to determine the main contributing factors to the microbiome diversity. OTUs common amongst all four sites were considered to be shared microbiota [6, 51].

## Results

### General differences

**Fig 2:**
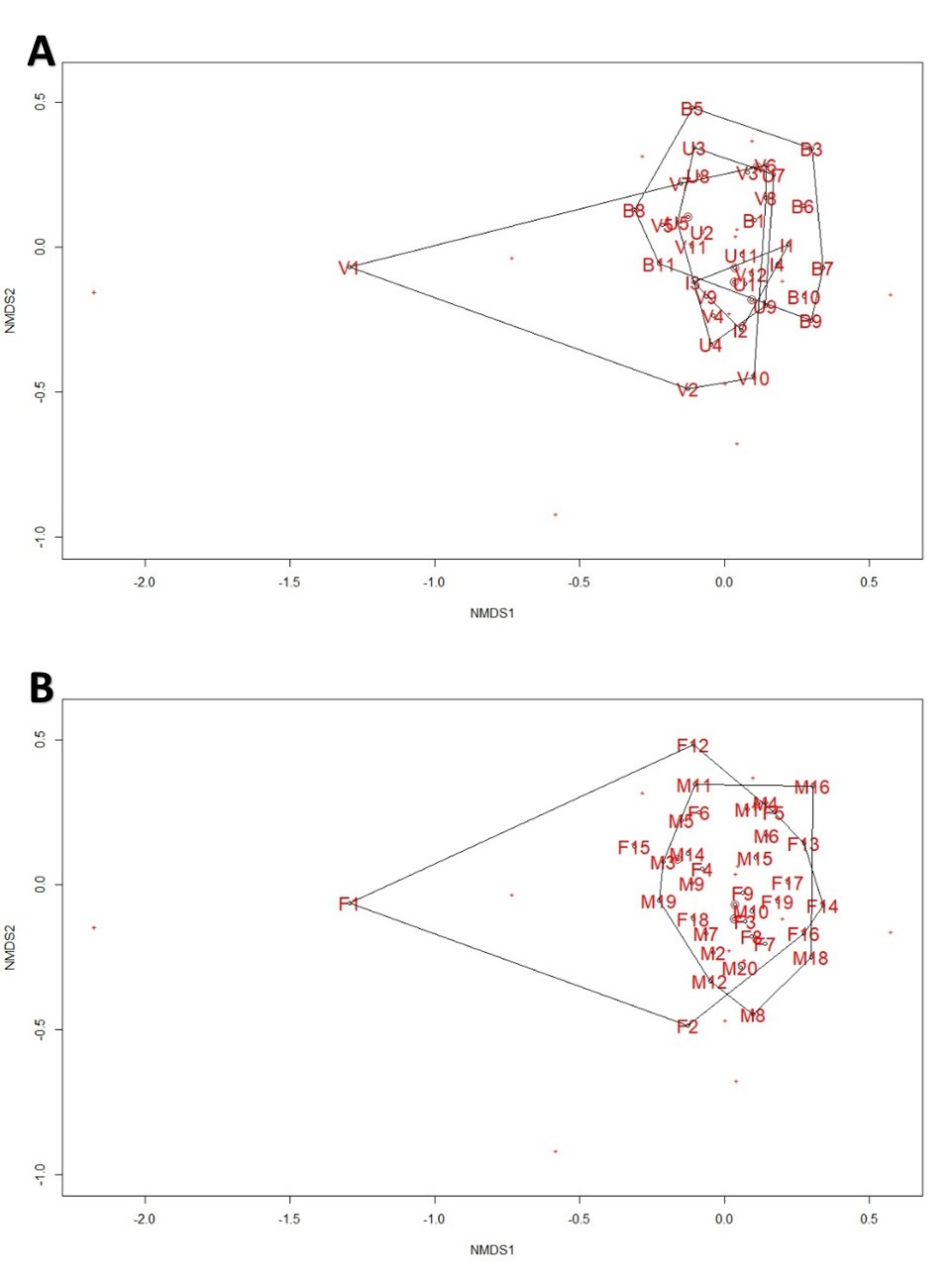
NMDS plots for the intestinal bacterium phyla in *Neoromicia nana* caught at wastewater treatment works (WWTW) and reference sites in KwaZulu-Natal, South Africa. (A) Individuals grouped by site (B = Buffelsdrift, I = Inkunzi, V = Verulam, U = Umbilo). (B) Individuals grouped by sex (M = Male, F = Female).

Sample size and sex ratio differed among sites (Verulam 10♂:2♀; Umbilo 4♂:8♀; Buffelsdrift 5♂:6♀; Inkunzi: 1♂:3♀). Although there were no significant differences in forearm length, body mass and BCI (all p > 0.05) among bats from different sites, females were significant larger than males in terms of forearm length, body mass and BCI (all p < 0.01). Bacterial diversity at all taxonomic levels did not correlate with BCI (all p > 0.05). NMDS of bacterial diversity shows large overlap among bats from different sites (Fig 2A) and both sexes (Fig 2B).

### Geographical differences

**Fig 3:**
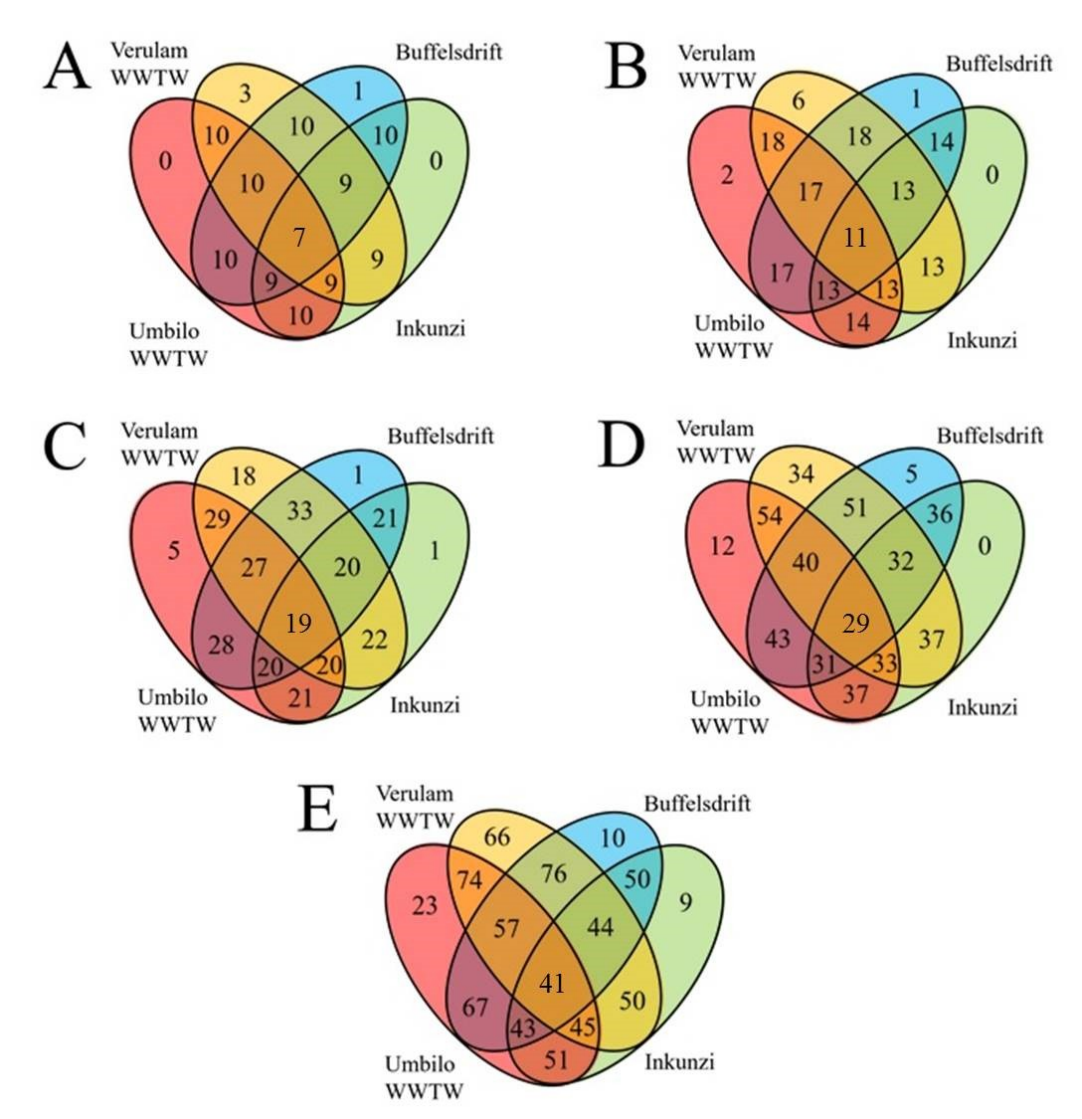
The total number of intestinal bacterial phyla (A), classes (B), orders (C), families (D) and genera (E) in *Neoromicia nana* caught at wastewater treatment works (WWTW) and reference sites in KwaZulu-Natal, South Africa. Green = Inkunzi, Blue = Buffelsdrift, Red = Umbilo WWTW and Orange = Verulam WWTW.

**Fig 4:**
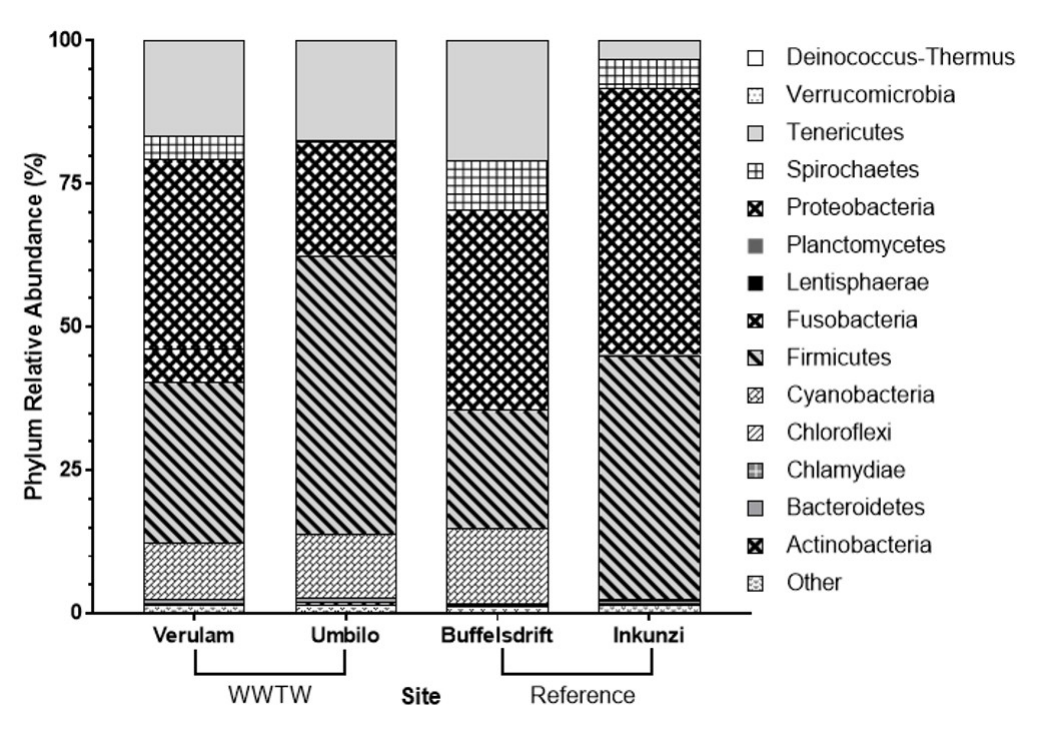
The average relative abundance of intestinal bacterium phyla in *Neoromicia nana* caught at wastewater treatment works (WWTW) and reference sites in KwaZulu-Natal, South Africa. “Other” includes OTUs not assigned to a phylum.

Bats captured at WWTW, specifically Verulam WWTW, showed the greatest microbiome diversity and the most unique OTUs (operational taxonomic units) at all taxonomic levels (Fig 3). This trend can also be seen in diversity indices, such that Verulam WWTW bats showed greater intestinal bacterial diversity than those from all other sites at each taxonomic level (Tables 1 and 2). Firmicutes and Proteobacteria were the two most abundant phyla found in bats, accounting for 20.5% to 48.6% and 19.9% to 46.6% of all intestinal bacterial diversity, respectively (Fig 4).

**Table 1:** Shannon-Weiner diversity index for intestinal bacteria in *Neoromicia nana* from WWTW and reference sites in KwaZulu-Natal, South Africa.

	Phylum	Class	Order	Family	Genus
Verulam	1.124	1.320	1.611	1.760	1.815
Umbilo	0.967	1.228	1.421	1.523	1.586
Buffelsdrift	0.988	1.263	1.404	1.449	1.477
Inkunzi	0.969	1.240	1.429	1.477	1.569

**Table 2:** Simpsons diversity index for intestinal bacteria in *Neoromicia nana* from WWTW and reference sites in KwaZulu-Natal, South Africa.

	Phylum	Class	Order	Family	Genus
Verulam	0.598 *	0.612	0.692	0.718	0.721
Umbilo	0.521 *	0.587	0.645	0.651	0.658
Buffelsdrift	0.535	0.623	0.646	0.648	0.650
Inkunzi	0.538	0.598	0.633	0.634	0.644

* significant difference (Z = 1.86 p = 0.031).

### The shared microbiota and factors influencing the microbial diversity

Because of the high diversity and variability of microbial assemblages, focus was placed on higher taxonomic levels and functional groups of the shared microbiota (7). As a result, eleven bacterial classes from seven phyla were identified as common among all sites (Table 3).

**Table 3:** The shared intestinal microbiota in *Neoromicia nana* caught at wastewater treatment works (WWTW) and reference sites in KwaZulu-Natal, South Africa. These microbes are common to all sites in this study.

Phylum	Class
Actinobacteria	Actinobacteria
Cyanobacteria	Chloroplast
Firmicutes	Bacilli
	Clostridia
Planctomycetes	Planctomycetia
Proteobacteria	Alphaproteobacteria
	Betaproteobacteria
	Epsilonproteobacteria
	Gammaproteobacteria
Spirochaetes	Spirochaetes
Tenericutes	Mollicutes

Among the factors tested (site, BCI and sex), site was the only significant predictor of microbial diversity at the genus level, explaining 18% of the variation among individuals (R2 = 0.18, F = 2.42, P = 0.002). OTU diversity, from phylum to family level, was significantly different between bats captured at Verulam WWTW and Umbilo WWTW (Table 4). Further, bats captured at Verulam WWTW had significantly higher OTU diversity than those captured at Inkunzi and Buffelsdrift reference sites (orders: χ2 = 9.68, df = 3, P = 0.02, Dunn’s Test z = -1.92, P = 0.03 and genera: χ2 = 4.24, df = 3, P = 0.24, Dunn’s Test z = -1.95, P = 0.03, respectively). Bats caught at Buffelsdrift reference site had more OTUs, at all taxonomic levels, in common with bats caught at WWTW than those caught at the Inkunzi reference site (Fig 3).

**Table 4:** The statistical differences among intestinal microbiota diversity in *Neoromicia nana* caught at wastewater treatment works (WWTW) and reference sites in KwaZulu-Natal, South Africa.

	χ2	P	Dunn’s Test P	z
Phyla	8.94	0.03	0.001	-2.99
Class	3.70	0.3	0.04	-1.73
Order	9.68	0.02	0.002	-2.95
Family	3.77	0.29	0.045	-1.70

df = 3.

### Significant differences in the shared microbiota

Bats captured at Umbilo had significantly fewer Spirochaetes than at other sites (P = 0.01, Dunn’s Test Verulam: P = 0.003, Buffelsdrift: P = 0.002 and Inkunzi: P = 0.019, S5 Table in S1 File). Proteobacteria were significantly more abundant in Inkunzi bats than those from Umbilo (P = 0.13, Dunn’s Test: P = 0.01). Within this phylum, Alphaproteobacteria were significantly more abundant in bats from Buffelsdrift than those from other sites (P = 0.07, Dunn’s Test Inkunzi: P = 0.01; Umbilo: P = 0.02; Verulam: P = 0.03), Epsilonproteobacteria were significantly more abundant in bats from Inkunzi than those from other sites (P = 0.08, Dunn’s Test Buffelsdrift: P<0.01; Umbilo: P = 0.02; Verulam: P = 0.02), and Gammaproteobacteria were significantly more abundant in bats from Inkunzi than those from Umbilo (P = 0.37, Dunn’s Test P = 0.04, S20 Table in S1 File). Firmicutes were significantly more abundant in bats from Umbilo (P = 0.02, Dunn’s Test Verulam: P = 0.02; Buffelsdrift: P = 0.003) and Inkunzi (P = 0.02, Dunn’s Test Buffelsdrift: P = 0.03, S9 Table in S1 File). Within this phylum, Bacilli abundance was significantly lower in bats from Buffelsdrift than those from other sites (P = 0.01, Dunn’s Test Inkunzi: P<0.01; Umbilo: P = 0.001; Verulam: P = 0.05) and Clostridia abundance was significantly lower in bats from Inkunzi than those from Buffelsdrift and Umbilo (P = 0.17, Dunn’s Test Buffelsdrift: P = 0.03 and Umbilo: P = 0.03, respectively, S9 Table in S1 File).

### Significant differences in other phyla

Bats at Verulam had significantly higher abundance of Chloroflexi (Buffelsdrift: P = 0.2, Dunn’s Test P = 0.037; Umbilo: P = 0.034) and Fusobacteria (P<0.001, Dunn’s Test Umbilo: P<0.001, Buffelsdrift: P<0.01 and Inkunzi: P<0.01, S4 Table in S1 File). Chlamydiae were significantly more abundant in Inkunzi bats (P = 0.19, Dunn’s Test Verulam: P = 0.024; Buffelsdrift P = 0.025, S3 Table in S1 File).

## Discussion

The putative shared microbiota of *N*. *nana*, defined by the OTUs shared among all sites (6,50), consists of seven phyla (Actinobacteria, Cyanobacteria, Firmicutes, Planctomycetes, Proteobacteria, Spirochaetes, Tenericutes) and eleven classes. Of these, all except Spirochaetes are typical for bats from all dietary strategies [52]. This suggests that the shared microbiota in these bats is highly conserved across geographic and phylogenetic distances.

Beyond the shared microbiota, location was the greatest driver of intestinal microbiome composition in *N*. *nana*. Great apes (3), and the external microbiomes of house flies, amphibians [51, 53, 54] and bats [55] show similar trends. Although OTU abundance and diversity may vary greatly among individuals from different sites, sympatric individuals from different species may share more similar intestinal microbiota than individuals of the same species who are separated geographically [3, 55]. This suggests that environmental factors and geography may be better predictors of the intestinal microbiome assemblages of *N*. *nana* than factors such as sex, host life-history and physiology, but do not influence the shared microbiota.

Several factors including sex, physiology, geography, diet, social interactions, exposure to chemical and biological pollutants and parasites [56–59] may influence species’ intestinal microbiomes. Location’s influence on the *N*. *nana*’s intestinal microbiome is probably mediated by the host’s association with WWTW; through exposure to wastewater [60], altered diet [60] and increased physiological stress [61], bats at WWTW harboured greater OTU diversity and more unique OTUs. Because the microbiome is so diverse, the effects that each microbial taxon experience may vary in response to environmental factors [59, 62], while the presence and abundance of certain taxa may have positive (pollution detoxification and breakdown of organic substances) or negative (pathogenicity) effects on the host’s fitness [63].

The high abundances of certain bacterial OTUs provide strong evidence for the transfer of bacteria from wastewater to bats foraging at these sites. For example, metal tolerant [60] filamentous bacteria of the phylum Chloroflexi, present in bats from Verulam WWTW, are often abundant in wastewater because they can remove biological nutrients [64]. These bats also harboured large numbers of the family Pirellulaceae (phylum: Planctomycetes) bacteria that are closely associated with WWTW due to their important role in nitrogen cycling [65] and their high resistance to ammonium, nitrite and nitrate concentrations [66]. Bats at Verulam WWTW also had significantly more Fusobacteria, typically found in large numbers in wastewater [67] and often linked to intestinal distress [68], intestinal inflammation, tumour formation and cancer of the mammalian GI tract [69].

Firmicutes, often found in wastewater, were one of the most abundant phyla found in *N*. *nana* individuals. These bacteria (particularly Lactobacillales), responsible for the synthesis of important metabolites involved in maintaining a healthy intestinal ecosystem [67], were abundant in bats at Verulam WWTW. This may be as a result of exercise-induce fatigue [70], evident by the high lactic acid concentrations previously reported in the pectoral muscles of WWTW bats [36, 71]. *Enterococcus*, another abundant wastewater-associated bacteria [31] in bats at Verulam WWTW, are responsible for the synthesis of vitamin K2, vitamin B12, folate, biotin [72] and enterocins (proteins that inhibit the growth of other bacteria) [73]. This suggests that bats at Verulam WWTW have high immunocompetence [73], perhaps to better cope with pathogen exposure. However, some bacteria belonging to this genus (e.g. *E*. *faecalis*) are linked with cancer promotion [69] through host chromosome instability and double-strand DNA breaks [74], infectious lesions, septicaemia, meningitis [75], diarrhoea [72] and increased gut permeability in mammals [76]. The liver is the body’s first defence against intestine-derived pathogens and receives 70% of its blood from the intestine. Because pathogens and pollutants may increase gut permeability, the combination of pollutant exposure and dysbiosis of the intestinal microbiota may cause translocation of gut microbes into the hepatic portal system [16], thereby resulting in an increased susceptibility to disease [68]. Bacteria, such as *Enterococcus*, may therefore have contributed to the DNA damage [18] and histopathological liver and kidney lesions in WWTW bats [30]. The high abundance of Clostridia in WWTW bats is further evidence for intestinal distress in these bats. These bacteria help regulate the immune system through the production of intestinal butyrate, a chemical that plays an important role in maintaining a healthy intestinal ecosystem by promoting colonic epithelial cell development and energy metabolism [77], and the induced production of T cells [78]. Butyrate, the preferred energy source for colonic cells, also has anti-inflammatory and anti-cancer properties [77].

The diets of bats associated with WWTW [25] may also significantly alter their intestinal microbiota assemblages. Specifically, abundant chironomid prey at WWTW [19, 79] is rich in chitin [80], and may favour the microbiota *Spironema* (phylum: Spirochaetes) and Chitinophagaceae (phylum: Bacteroidetes). These bacteria are responsible for fibre digestion, short-chain fatty acid production [61], as well as the breakdown of chitin [81] and complex polysaccharides [82]. Further, polyunsaturated fatty acid (PUFA) [29, 83] rich chironomid diets may alter Tenericutes abundance [62, 84], a phylum containing both commensal and parasitic bacteria [85].

Many of the bacteria associated with the WWTW bats can cause histopathological lesions [84, 86], thus lesions in the detoxification organs of *N*. *nana* caught at WWTW [30] should be excised and sequenced to investigate possible links between these bacteria and lesion formation. Additionally, bacterial assemblages in the water and emerging insects [87] at WWTW should be studied to determine the routes pathogens pass from wastewater to these bats.

## Conclusion

The seven phyla and eleven classes shared by the individuals captured in this study may comprise the potential core intestinal microbiome of the insectivorous bat, *N*. *nana*. However, establishing spatial and temporal consistency of shared microbiota is necessary to validate the core microbial assemblage composition. Variation in *N*. *nana*’s microbiome appears to be driven by geography, and further exacerbated by their association with WWTW. Concurrent stressors *in N*. *nana* at WWTW were reported before by our group and include altered diets, toxicant exposure, increased lactic acid production [19, 29, 30, 36] may cause dysbiosis of gastro-intestinal assemblages [63]. This, in turn, may affect the host’s metabolism [82], immune function [78, 88] and behaviour [89] and warrant further investigation. However, the altered abundances of bacteria, such as Chitinophagaceae, in WWTW bats points towards an adaptive microbial assemblage. Despite the deleterious impact’s concomitant stressors, such as altered diet and toxicant exposure, associated with foraging at WWTWs have on these bats, our findings suggest that the intestinal microbiome of *N*. *nana* can cope through changes in assemblage composition.

## Supporting information

S1 File (DOCX)

S1 Data (XLSX)

S2 Data (XLSX)

S3 Data (XLSX)
