# Supplementary material for: Wastewater treatment works change the intestinal microbiomes of insectivorous bats
Source: PLoS One. 2021 Mar 3;16(3):e0247475. doi: 10.1371/journal.pone.0247475 (PMC7928523; doi:10.1371/journal.pone.0247475)
Supplement: S1 File — (DOCX) [file pone.0247475.s001.docx]

**Supplementary material**

**Mehl et al,** **Wastewater treatment works change the intestinal microbiomes of insectivorous bats.**

Results

S1 Table 1: Statistically significant differences in intestinal Actinobacteria abundances observed among *Neoromicia nana* caught at wastewater treatment works (WWTW) and reference sites in KwaZulu-Natal, South Africa.

| Sites | | K-W rank sums Test | | | Dunn's Test | |
| --- | --- | --- | --- | --- | --- | --- |
|  |  | χ^2^ | df | P | z | P |
| **Phylum Actinobacteria** | | | | | | |
| Class Actinobacteria | | | | | | |
| Order Actinomycetales | | | | | | |
| Family Dermacoccaceae | | | | | | |
| Genus *Dermacoccus* | | | | | | |
| Buffelsdrift | Verulam WWTW | 5.22 | 3 | 0.16 | -1.95 | 0.026 |
|  | Umbilo WWTW |  |  |  | -1.95 | 0.026 |
| Family Nocardioidaceae | | | | | | |
| Inkunzi | Buffelsdrift | 4.45 | 3 | 0.22 | -1.87 | 0.031 |
|  | Verulam WWTW |  |  |  | 1.89 | 0.03 |
| Family Micrococcaceae | | | | | | |
| Inkunzi | Umbilo WWTW | 4.61 | 3 | 0.2 | 1.89 | 0.03 |
|  | Verulam WWTW |  |  |  | 1.89 | 0.03 |
| Genus *Mycobacterium* | | | | | | |
| Inkunzi | Umbilo WWTW | 4.94 | 3 | 0.18 | 1.99 | 0.024 |
|  | Verulam WWTW |  |  |  | 1.99 | 0.024 |
| Genus *Microbispora* | | | | | | |
| Inkunzi | Umbilo WWTW | 4.61 | 3 | 0.2 | 1.89 | 0.03 |
|  | Verulam WWTW |  |  |  | 1.89 | 0.03 |
| Genus *Microbacterium* | | | | | | |
| Inkunzi | Buffelsdrift | 4.45 | 3 | 0.22 | -1.87 | 0.03 |
|  | Verulam WWTW |  |  |  | 1.89 | 0.03 |

S1 Table 2: Statistically significant differences in intestinal Cyanobacteria abundances observed among *Neoromicia nana* caught at wastewater treatment works (WWTW) and reference sites in KwaZulu-Natal, South Africa.

| Sites | | K-W rank sums Test | | | Dunn's Test | |  |
| --- | --- | --- | --- | --- | --- | --- | --- |
|  |  | χ^2^ | df | P | χ^2^ | df |  |
| **Phylum Cyanobacteria** | | | | | | |  |
| Class Chloroplast | | | | | | |  |
| Order Stramenopiles | | | | | | |  |
| Verulam WWTW | Inkunzi | 4.85 | 3 | 0.18 | -1.86 | 0.03 |  |
|  | Umbilo WWTW |  |  |  | -1.76 | 0.04 |  |

S1 Table 3: Statistically significant differences in intestinal Chlamydiae abundances observed among *Neoromicia nana* caught at wastewater treatment works (WWTW) and reference sites in KwaZulu-Natal, South Africa.

| Sites | | K-W rank sums Test | | | | | | Dunn's Test | | | |  | |  | |
| --- | --- | --- | --- | --- | --- | --- | --- | --- | --- | --- | --- | --- | --- | --- | --- |
|  |  | χ^2^ | | df | | P | | χ^2^ | | df | |  | |  | |
| **Phylum Chlamydiae** | | | | | | | | | | | | |  |  |  |
| Inkunzi | Verulam WWTW | | 4.79 | | 3 | | 0.19 | | -1.99 | | 0.024 | |  | |  |
|  | Buffelsdrift | |  |  |  |  |  |  | -1.97 | | 0.025 | |  | |  |

S1 Table 4: Statistically significant differences in intestinal Chloroflexi abundances observed among *Neoromicia nana* caught at wastewater treatment works (WWTW) and reference sites in KwaZulu-Natal, South Africa.

| Sites | | K-W rank sums Test | | | | | | Dunn's Test | | | | |  |
| --- | --- | --- | --- | --- | --- | --- | --- | --- | --- | --- | --- | --- | --- |
|  |  | χ^2^ | | df | | P | | χ^2^ | | df | | |  |
| **Phylum Chloroflexi** | | | | | | | | | | | |  |  |
| Verulam WWTW | Buffelsdrift | | 4.62 | | 3 | | 0.2 | | 1.79 | | 0.037 |  |  |
|  | Umbilo WWTW | |  |  |  |  |  |  | 1.83 | | 0.034 |  |  |

S1 Table 5: Statistically significant differences in intestinal Spirochaetes abundances observed among *Neoromicia nana* caught at wastewater treatment works (WWTW) and reference sites in KwaZulu-Natal, South Africa.

| Sites | | K-W rank sums Test | | | | | | Dunn's Test | | | | |  |
| --- | --- | --- | --- | --- | --- | --- | --- | --- | --- | --- | --- | --- | --- |
|  |  | χ^2^ | | df | | P | | χ^2^ | | df | | |  |
| **Phylum Spirochaetes** | | | | | | | | | | | |  |  |
| Reference | WWTW | | 246* | | 38 | | 0.047 | |  | |  |  |  |
| Verulam WWTW | Umbilo WWTW | | 11.71 | | 3 | | 0.01 | | 2.77 | | 0.003 |  |  |
| Buffelsdrift |  |  |  |  |  |  |  |  | 2.96 | | 0.002 |  |  |
| Inkunzi |  |  |  |  |  |  |  |  | -2.08 | | 0.02 |  |  |

* indicate W-value

S1 Table 6: Statistically significant differences in intestinal Fusobacteria abundances observed among *Neoromicia nana* caught at wastewater treatment works (WWTW) and reference sites in KwaZulu-Natal, South Africa.

| Sites | | K-W rank sums Test | | | | | | Dunn's Test | | | | |  |
| --- | --- | --- | --- | --- | --- | --- | --- | --- | --- | --- | --- | --- | --- |
|  |  | χ^2^ | | df | | P | | χ^2^ | | df | | |  |
| **Phylum Fusobacteria** | | | | | | | | | | | |  |  |
| Verulam WWTW | Umbilo WWTW | | 13.89 | | 3 | | 0.0004 | | 3.36 | | 0.0004 |  |  |
|  | Buffelsdrift | |  |  |  |  |  |  | 2.4 | | 0.008 |  |  |
|  | Inkunzi | |  |  |  |  |  |  | 2.6 | | 0.005 |  |  |

S1 Table 7: Statistically significant differences in intestinal Tenericutes abundances observed among *Neoromicia nana* caught at wastewater treatment works (WWTW) and reference sites in KwaZulu-Natal, South Africa.

| Sites | | K-W rank sums Test | | | | | | Dunn's Test | | | |  |
| --- | --- | --- | --- | --- | --- | --- | --- | --- | --- | --- | --- | --- |
|  |  | χ^2^ | | df | | P | | χ^2^ | | df | |  |
| **Phylum Tenericutes** | | | | | | | | | | | |  |
| Class Mollicutes | | | | | | | | | | | |  |
| Order Mycoplasmatales | | | | | | | | | | | |  |
| Family Mycoplasmataceae | | | | | | | | | | | |  |
| Genus *Ureaplasma* | | | | | | | | | | | |  |
| Buffelsdrift | Verulam WWTW | | 3.11 | | 3 | | 0.38 | | 1.66 | | 0.05 |  |

S1 Table 8: Statistically significant differences in intestinal Planctomycetes abundances observed among *Neoromicia nana* caught at wastewater treatment works (WWTW) and reference sites in KwaZulu-Natal, South Africa.

| Sites | | K-W rank sums Test | | | | | | Dunn's Test | | | |  |
| --- | --- | --- | --- | --- | --- | --- | --- | --- | --- | --- | --- | --- |
|  |  | χ^2^ | | df | | P | | χ^2^ | | df | |  |
| **Phylum Planctomycetes** | | | | | | | | | | | |  |
| Class Planctomycetia | | | | | | | | | | | |  |
| Order Pirellulales | | | | | | | | | | | |  |
| Family Pirellulaceae | | | | | | | | | | | |  |
| Verulam WWTW | Umbilo WWTW | | 3.17 | | 3 | | 0.24 | | -1.84 | | 0.03 |  |
| Class Planctomycetia | | | | | | | | | | | |  |
| Order Gemmatales | | | | | | | | | | | |  |
| Family Gemmataceae | | | | | | | | | | | |  |
| Genus *Gemmata* | | | | | | | | | | | |  |
| Verulam WWTW | Buffelsdrift | | 4.62 | | 3 | | 0.2 | | -1.79 | | 0.04 |  |
|  | Inkunzi | |  |  |  |  |  |  | -1.83 | | 0.03 |  |

S1 Table 9: Statistically significant differences in intestinal Firmicutes abundances observed among *Neoromicia nana* caught at wastewater treatment works (WWTW) and reference sites in KwaZulu-Natal, South Africa.

| Sites | | K-W rank sums Test | | | Dunn's Test | |  | |
| --- | --- | --- | --- | --- | --- | --- | --- | --- |
|  |  | χ^2^ | df | P | χ^2^ | df |  | |
| **Phylum Firmicutes** | | | | | | | |  |
| Umbilo WWTW | Verulam WWTW | 9.55 | 3 | 0.02 | -1.99 | 0.02 | |  |
|  | Buffelsdrift |  |  |  | 2.8 | 0.003 | |  |
| Inkunzi | Buffelsdrift | 9.55 | 3 | 0.02 | -1.9 | 0.03 | |  |
| Class Clostridia | | | | | | | |  |
| Buffelsdrift | Inkunzi | 4.97 | 3 | 0.17 | 1.94 | 0.02 | |  |
| Umbilo WWTW | Inkunzi |  |  |  | -1.93 | 0.03 | |  |
| Order OPB54 | | | | | | | |  |
| Verulam WWTW | Buffelsdrift | 4.62 | 3 | 0.2 | -1.79 | 0.04 | |  |
|  | Umbilo WWTW |  |  |  | -1.83 | 0.04 | |  |
| Order Clostridiales | | | | | | | |  |
| Buffelsdrift | Inkunzi | 5.21 | 3 | 0.16 | 1.95 | 0.03 | |  |
| Umbilo WWTW | Inkunzi |  |  |  | -1.93 | 0.03 | |  |
| Family Clostridiaceae | | | | | | | |  |
| Buffelsdrift | Inkunzi | 4.98 | 3 | 0.17 | 1.75 | 0.04 | |  |
|  | Verulam WWTW |  |  |  | 1.92 | 0.03 | |  |
| Genus *Candidatus arthromitus* | | | | | | | |  |
| Buffelsdrift | Verulam WWTW | 6.44 | 3 | 0.09 | 2.43 | 0.008 | |  |
| Class Bacilli | | | | | | | |  |
| Inkunzi | Buffelsdrift | 11.38 | 3 | 0.01 | -2.39 | 0.009 | |  |
| Umbilo WWTW | Buffelsdrift |  |  |  | -3.09 | 0.001 | |  |
| Verulam WWTW | Buffelsdrift |  |  |  | -1.69 | 0.05 | |  |
| Order Bacillales | | | | | | | |  |
| Inkunzi | Buffelsdrift | 12.9 | 3 | <0.0001 | -2.52 | 0.006 | |  |
|  | Verulam WWTW |  |  |  | 1.89 | 0.03 | |  |
| Umbilo WWTW | Buffelsdrift |  |  |  | -3.06 | 0.001 | |  |
|  | Verulam WWTW |  |  |  | 2.18 | 0.01 | |  |
| Family Bacillaceae | | | | | | | |  |
| Inkunzi | Buffelsdrift | 13.09 | 3 | <0.0001 | -2.56 | 0.005 | |  |
|  | Verulam WWTW |  |  |  | 1.94 | 0.03 | |  |
| Umbilo WWTW | Buffelsdrift |  |  |  | -3.06 | 0.001 | |  |
|  | Verulam WWTW |  |  |  | 2.2 | 0.01 | |  |
| Genus *Bacillus* | | | | | | | |  |
| Inkunzi | Buffelsdrift | 13.28 | 3 | <0.0001 | -2.54 | 0.006 | |  |
|  | Verulam WWTW |  |  |  | 1.9 | 0.03 | |  |
| Umbilo WWTW | Buffelsdrift |  |  |  | -3.11 | 0.0009 | |  |
|  | Verulam WWTW |  |  |  | 2.24 | 0.01 | |  |
| Genus *Marinibacillus* | | | | | | | |  |
| Inkunzi | Buffelsdrift | 12.27 | 3 | 0.01 | -3.25 | 0.0006 | |  |
|  | Umbilo WWTW |  |  |  | 3.29 | 0.0005 | |  |
|  | Verulam WWTW |  |  |  | 2.78 | 0.003 | |  |
| Family Listeriaceae | | | | | | | |  |
| Umbilo WWTW | Buffelsdrift | 4.62 | 3 | 0.2 | -1.79 | 0.04 | |  |
|  | Verulam WWTW |  |  |  | 1.83 | 0.03 | |  |
| Family Staphylococcaceae | | | | | | | |  |
| Reference | WWTW | 246* | 38 | 0.01 |  |  | |  |
| Buffelsdrift | Umbilo WWTW | 9.12 | 3 | 0.03 | 2.98 | 0.002 | |  |
|  | Verulam WWTW |  |  |  | 1.99 | 0.02 | |  |
| Genus *Staphylococcus* | | | | | | | |  |
| Reference | WWTW | 245* | 38 | 0.01 |  |  | |  |
| Buffelsdrift | Umbilo WWTW | 8.76 | 3 | 0.03 | 2.92 | 0.002 | |  |
|  | Verulam WWTW |  |  |  | 1.9 | 0.03 | |  |
| Family Planococcaceae | | | | | | | |  |
| Genus *Planomicrobium* | | | | | | | |  |
| Inkunzi | Buffelsdrift | 8.75 | 3 | 0.03 | -2.67 | 0.004 | |  |
|  | Umbilo WWTW |  |  |  | 2.7 | 0.003 | |  |
|  | Verulam WWTW |  |  |  | 2.7 | 0.003 | |  |
| Genus *Planococcus* | | | | | | | |  |
| Inkunzi | Buffelsdrift | 8.75 | 3 | 0.03 | -2.67 | 0.004 | |  |
|  | Umbilo WWTW |  |  |  | 2.7 | 0.003 | |  |
|  | Verulam WWTW |  |  |  | 2.7 | 0.003 | |  |
| Class Bacilli | | | | | | | |  |
| Order Lactobacillales | | | | | | | |  |
| WWTW | Reference | 74* | 38 | 0.002 |  |  | |  |
| Verulam WWTW | Buffelsdrift | 10.18 | 3 | 0.02 | -2.95 | 0.002 | |  |
|  | Inkunzi |  |  |  | -1.76 | 0.04 | |  |
| Umbilo WWTW | Buffelsdrift |  |  |  | -2.15 | 0.02 | |  |
| Family Aerococcaceae | | | | | | | |  |
| WWTW | Reference | 57* | 38 | 0.0001 |  |  | |  |
| Verulam WWTW | Buffelsdrift | 15.38 | 3 | <0.0001 | -3.01 | 0.001 | |  |
|  | Inkunzi |  |  |  | -1.68 | 0.05 | |  |
| Umbilo WWTW | Buffelsdrift |  |  |  | -3.45 | 0.0003 | |  |
|  | Inkunzi |  |  |  | -2 | 0.02 | |  |
| Genus *Facklamia* | | | | | | | |  |
| WWTW | Reference | 65.5* | 38 | 0.0003 |  |  | |  |
| Verulam WWTW | Buffelsdrift | 14.03 | 3 | <0.0001 | -2.77 | 0.003 | |  |
| Umbilo WWTW | Buffelsdrift |  |  |  | -3.39 | 0.0003 | |  |
|  | Inkunzi |  |  |  | -1.91 | 0.03 | |  |
| Family Carnobacteriaceae | | | | | | | |  |
| WWTW | Reference | 58.5* | 38 | 0.0004 |  |  | |  |
| Verulam WWTW | Buffelsdrift | 12.6 | 3 | 0.01 | -2.88 | 0.002 | |  |
|  | Inkunzi |  |  |  | -2.01 | 0.02 | |  |
| Umbilo WWTW | Buffelsdrift |  |  |  | -2.77 | 0.003 | |  |
|  | Inkunzi |  |  |  | -1.93 | 0.03 | |  |
| Genus *Granulicatella* | | | | | | | |  |
| Verulam WWTW | Buffelsdrift | 4.62 | 3 | 0.2 | -1.79 | 0.04 | |  |
|  | Umbilo WWTW |  |  |  | -1.83 | 0.03 | |  |
| Genus *Trichococcus* | | | | | | | |  |
| WWTW | Reference | 126* | 28 | 0.04 |  |  | |  |
| Umbilo WWTW | Buffelsdrift | 5.3 | 3 | 0.15 | -1.83 | 0.03 | |  |
| Verulam WWTW | Buffelsdrift |  |  |  | -2.15 | 0.02 | |  |
| Family Enterococcaceae | | | | | | | |  |
| Males | Females | 85* | 38 | 0.003 |  |  | |  |
| Verulam WWTW | Umbilo WWTW | 4.62 | 3 | 0.2 | -2.08 | 0.02 | |  |
| Genus *Enterococcus* | | | | | | | |  |
| Males | Females | 85* | 38 | 0.003 |  |  | |  |
| Verulam WWTW | Umbilo WWTW | 4.41 | 3 | 0.22 | -2.05 | 0.02 | |  |
| Genus *Vagococcus* | | | | | | | |  |
| Males | Females | 111* | 38 | 0.02 |  |  | |  |
| Inkunzi | Buffelsdrift | 4.57 | 3 | 0.24 | -1.71 | 0.04 | |  |
| Family *Streptococcaceae* | | | | | | | |  |
| Inkunzi | Umbilo WWTW | 7.22 | 3 | 0.07 | 1.77 | 0.04 | |  |
| Verulam WWTW | Umbilo WWTW |  |  |  | -2.43 | 0.008 | |  |
| Genus *Lactococcus* | | | | | | | |  |
| Inkunzi | Buffelsdrift | 12.27 | 3 | 0.01 | -3.25 | 0.0006 | |  |
|  | Umbilo WWTW |  |  |  | 3.29 | 0.0005 | |  |
|  | Verulam WWTW |  |  |  | 2.78 | 0.003 | |  |
| Genus *Streptococcus* | | | | | | | |  |
| Verulam WWTW | Inkunzi | 6.05 | 3 | 0.11 | -1.83 | 0.03 | |  |
|  | Umbilo WWTW |  |  |  | -2.18 | 0.01 | |  |
| Class Bacilli | | | | | | | |  |
| Order Turicibacterales | | | | | | | |  |
| Inkunzi | Buffelsdrift | 8.75 | 3 | 0.03 | -2.67 | 0.004 | |  |
|  | Umbilo WWTW |  |  |  | 2.7 | 0.003 | |  |
|  | Verulam WWTW |  |  |  | 2.7 | 0.003 | |  |

* indicate W-value

S1 Table 20: Statistically significant differences in intestinal Proteobacteria abundances observed among *Neoromicia nana* caught at wastewater treatment works (WWTW) and reference sites in KwaZulu-Natal, South Africa.

| Sites | | K-W rank sums Test | | | | | | Dunn's Test | | | |  |
| --- | --- | --- | --- | --- | --- | --- | --- | --- | --- | --- | --- | --- |
|  |  | χ^2^ | | df | | P | | χ^2^ | | df | |  |
| **Phylum Proteobacteria** | | | | | | | | | | | |  |
| Inkunzi | Umbilo WWTW | | 5.69 | | 3 | | 0.13 | | -2.28 | | 0.01 |  |
| Class Alphaproteobacteria | | | | | | | | | | | |  |
| Buffelsdrift | Verulam WWTW | | 6.92 | | 3 | | 0.07 | | 1.82 | | 0.03 |  |
|  | Umbilo WWTW | |  |  |  |  |  |  | 2.05 | | 0.02 |  |
|  | Inkunzi | |  |  |  |  |  |  | 2.21 | | 0.01 |  |
| Order Caulobacterales | | | | | | | | | | | |  |
| Inkunzi | Verulam WWTW | | 11.98 | | 3 | | 0.01 | | 2.73 | | 0.003 |  |
|  | Umbilo WWTW | |  |  |  |  |  |  | 3.25 | | 0.0006 |  |
|  | Buffelsdrift | |  |  |  |  |  |  | -3.21 | | 0.0007 |  |
| Family Caulobacteraceae | | | | | | | | | | | |  |
| Genus *Phenylobacterium* | | | | | | | | | | | |  |
| Inkunzi | Verulam WWTW | | 8.75 | | 3 | | 0.03 | | 2.7 | | 0.003 |  |
|  | Umbilo WWTW | |  |  |  |  |  |  | 2.7 | | 0.003 |  |
|  | Buffelsdrift | |  |  |  |  |  |  | -2.67 | | 0.004 |  |
| Genus *Caulobacter* | | | | | | | | | | | |  |
| Inkunzi | Verulam WWTW | | 8.75 | | 3 | | 0.03 | | 2.7 | | 0.003 |  |
|  | Umbilo WWTW | |  |  |  |  |  |  | 2.7 | | 0.004 |  |
|  | Buffelsdrift | |  |  |  |  |  |  | -2.67 | | 0.004 |  |
| Genus *Brevundimonas* | | | | | | | | | | | |  |
| Inkunzi | Umbilo WWTW | | 4.62 | | 3 | | 0.2 | | 1.94 | | 0.03 |  |
|  | Buffelsdrift | |  |  |  |  |  |  | -1.92 | | 0.03 |  |
| Order Rhizobiales | | | | | | | | | | | |  |
| Verulam WWTW | Umbilo WWTW | | 3.9 | | 3 | | 0.27 | | 1.97 | | 0.02 |  |
| Order Rhodobacterales | | | | | | | | | | | |  |
| Verulam WWTW | Umbilo WWTW | | 4.31 | | 3 | | 0.23 | | -1.9 | | 0.03 |  |
| Order Sphingomonadales | | | | | | | | | | | |  |
| Verulam WWTW | Umbilo WWTW | | 4.13 | | 3 | | 0.25 | | -1.95 | | 0.03 |  |
| Order Rickettsiales | | | | | | | | | | | |  |
| Verulam WWTW | Inkunzi | | 15.52 | | 3 | | <0.0001 | | -2.09 | | 0.02 |  |
| Buffelsdrift | Verulam WWTW | |  |  |  |  |  |  | 1.68 | | 0.05 |  |
|  | Umbilo WWTW | |  |  |  |  |  |  | 3.2 | | 0.0007 |  |
|  | Inkunzi | |  |  |  |  |  |  | 3.27 | | 0.0005 |  |
| Order Sphingomonadales | | | | | | | | | | | |  |
| Family Sphingomonadaceae | | | | | | | | | | | |  |
| Genus *Sphingomonas* | | | | | | | | | | | |  |
| Verulam WWTW | Umbilo WWTW | | 4.36 | | 3 | | 0.23 | | -1.95 | | 0.03 |  |
| Order Rhizobiales | | | | | | | | | | | |  |
| Family Bartonellaceae | | | | | | | | | | | |  |
| Genus *Bartonella* | | | | | | | | | | | |  |
| Inkunzi | Buffelsdrift | | 9.4 | | 3 | | 0.02 | | -2.46 | | 0.007 |  |
|  | Verulam WWTW | |  |  |  |  |  |  | 2.48 | | 0.007 |  |
| Umbilo WWTW | Buffelsdrift | |  |  |  |  |  |  | -1.79 | | 0.04 |  |
|  | Verulam WWTW | |  |  |  |  |  |  | 1.83 | | 0.03 |  |
| Family Bradyrhizobiaceae | | | | | | | | | | | |  |
| Inkunzi | Verulam WWTW | | 4.06 | | 3 | | 0.25 | | 1.65 | | 0.05 |  |
| Genus *Bosea* | | | | | | | | | | | |  |
| Umbilo WWTW | Buffelsdrift | | 4.62 | | 3 | | 0.2 | | -1.79 | | 0.04 |  |
|  | Verulam WWTW | |  |  |  |  |  |  | 1.83 | | 0.04 |  |
| Family Phyllobacteriaceae | | | | | | | | | | | |  |
| Verulam WWTW | Buffelsdrift | | 4.62 | | 3 | | 0.2 | | -1.79 | | 0.04 |  |
|  | Umbilo WWTW | |  |  |  |  |  |  | 0.04 | | 0.04 |  |
| Family Hyphomicrobiaceae | | | | | | | | | | | |  |
| Genus *Hyphomicrobium* | | | | | | | | | | | |  |
| Verulam WWTW | Umbilo WWTW | | 6 | | 3 | | 0.11 | | -2.28 | | 0.01 |  |
| Genus *Rhodoplanes* | | | | | | | | | | | |  |
| Umbilo WWTW | Inkunzi | | 7.01 | | 3 | | 0.07 | | -1.65 | | 0.05 |  |
|  | Verulam WWTW | |  |  |  |  |  |  | 2.33 | | 0.01 |  |
| Buffelsdrift | Verulam WWTW | |  |  |  |  |  |  | 1.73 | | 0.04 |  |
| Family Rhizobiaceae | | | | | | | | | | | |  |
| Genus *Agrobacterium* | | | | | | | | | | | |  |
| Umbilo WWTW | Inkunzi | | 3.82 | | 3 | | 0.28 | | -1.75 | | 0.04 |  |
| Genus *Rhizobium* | | | | | | | | | | | |  |
| Umbilo WWTW | Buffelsdrift | | 4.17 | | 3 | | 0.24 | | 1.7 | | 0.05 |  |
| Class Betaproteobacteria | | | | | | | | | | | |  |
| Order Methylophilales | | | | | | | | | | | |  |
| Males | Females | | 142.5* | | 38 | | 0.02 | |  | |  |  |
| Class Epsilonproteobacteria | | | | | | | | | | | |  |
| Inkunzi | Verulam WWTW | | 6.92 | | 3 | | 0.07 | | 1.98 | | 0.02 |  |
|  | Umbilo WWTW | |  |  |  |  |  |  | 2.08 | | 0.02 |  |
|  | Buffelsdrift | |  |  |  |  |  |  | -2.6 | | 0.005 |  |
| Order Campylobacterales | | | | | | | | | | | |  |
| Family Helicobacteraceae | | | | | | | | | | | |  |
| Genus *Helicobacter* | | | | | | | | | | | |  |
| Inkunzi | Buffelsdrift | | 5.29 | | 3 | | 0.15 | | -2.21 | | 0.01 |  |
| Genus *Flexispira* | | | | | | | | | | | |  |
| Inkunzi | Verulam WWTW | | 13.68 | | 3 | | <0.0001 | | 2.94 | | 0.002 |  |
|  | Umbilo WWTW | |  |  |  |  |  |  | 3.13 | | 0.001 |  |
|  | Buffelsdrift | |  |  |  |  |  |  | -3.64 | | 0.0001 |  |
| Class Gammaproteobacteria | | | | | | | | | | | |  |
| Order Legionellales | | | | | | | | | | | |  |
| Verulam WWTW | Buffelsdrift | | 10.53 | | 3 | | 0.01 | | -2.73 | | 0.003 |  |
|  | Umbilo WWTW | |  |  |  |  |  |  | -2.79 | | 0.003 |  |
| Family Legionellaceae | | | | | | | | | | | |  |
| Verulam WWTW | Buffelsdrift | | 7.11 | | 3 | | 0.07 | | -2.22 | | 0.01 |  |
|  | Umbilo WWTW | |  |  |  |  |  |  | -2.27 | | 0.01 |  |
| Family Coxiellaceae | | | | | | | | | | | |  |
| Genus *Rickettsiella* | | | | | | | | | | | |  |
| Inkunzi | Verulam WWTW | | 8.75 | | 3 | | 0.03 | | 2.7 | | 0.003 |  |
|  | Umbilo WWTW | |  |  |  |  |  |  | 2.7 | | 0.003 |  |
|  | Buffelsdrift | |  |  |  |  |  |  | -2.67 | | 0.004 |  |

* indicate W-value

S1 Table 31: Statistically significant differences in intestinal Bacteroidetes abundances observed among *Neoromicia nana* caught at wastewater treatment works (WWTW) and reference sites in KwaZulu-Natal, South Africa.

| Sites | | K-W rank sums Test | | | | | | Dunn's Test | | | |  |
| --- | --- | --- | --- | --- | --- | --- | --- | --- | --- | --- | --- | --- |
|  |  | χ^2^ | | df | | P | | χ^2^ | | df | |  |
| **Phylum Bacteroidetes** | | | | | | | | | | | |  |
| Class Bacteroidia | | | | | | | | | | | |  |
| Verulam WWTW | Buffelsdrift | | 4.2 | | 3 | | 0.24 | | -1.9 | | 0.03 |  |
| Order Bacteroidales | | | | | | | | | | | |  |
| Family Porphyromonadaceae | | | | | | | | | | | |  |
| Verulam WWTW | Buffelsdrift | | 4.62 | | 3 | | 0.2 | | -1.79 | | 0.04 |  |
|  | Umbilo WWTW | |  |  |  |  |  |  | -1.83 | | 0.04 |  |
| Class Sphingobacteriia | | | | | | | | | | | |  |
| Order Sphingobacteriales | | | | | | | | | | | |  |
| Family Sphingobacteriaceae | | | | | | | | | | | |  |
| Genus *Sphingobacterium* | | | | | | | | | | | |  |
| Inkunzi | Umbilo WWTW | | 4.94 | | 3 | | 0.18 | | 1.99 | | 0.02 |  |
|  | Verulam WWTW | |  |  |  |  |  |  | 1.99 | | 0.02 |  |
| Class Saprospirae | | | | | | | | | | | |  |
| Verulam WWTW | Buffelsdrift | | 7.11 | | 3 | | 0.07 | | -2.22 | | 0.01 |  |
|  | Umbilo WWTW | |  |  |  |  |  |  | -2.27 | | 0.01 |  |
| Order Saprospirales | | | | | | | | | | | |  |
| Family Chitinophagaceae | | | | | | | | | | | |  |
| Genus *Sediminibacterium* | | | | | | | | | | | |  |
| Verulam WWTW | Buffelsdrift | | 4.62 | | 3 | | 0.2 | | -1.79 | | 0.04 |  |
|  | Umbilo WWTW | |  |  |  |  |  |  | -1.83 | | 0.04 |  |
